# A Prospective Study of Azilsartan Medoxomil in the Treatment of Patients with Essential Hypertension and Type 2 Diabetes in Asia

**DOI:** 10.1155/2022/2717291

**Published:** 2022-01-07

**Authors:** Chaicharn Deerochanawong, Kuan-Cheng Chang, Yu Cho Woo, Wen-Ter Lai, Aurauma Chutinet

**Affiliations:** ^1^Department of Medicine, Rajavithi Hospital, College of Medicine, Rangsit Medical School, Bangkok, Thailand; ^2^Division of Cardiovascular Medicine, Department of Medicine, China Medical University Hospital and Graduate Institute of Biomedical Sciences, China Medical University, Taichung, Taiwan; ^3^Department of Medicine, Queen Mary Hospital, Pok Fu Lam, Hong Kong; ^4^Cardiology, Kaohsiung Medical University Chung-Ho Memorial Hospital, Kaohsiung, Taiwan; ^5^Chulalongkorn Stroke Center, King Chulalongkorn Memorial Hospital, Thai Red Cross Society, Department of Medicine, Faculty of Medicine, Chulalongkorn University, Bangkok, Thailand

## Abstract

This phase 4 study evaluated the efficacy and safety of azilsartan medoxomil (AZL-M) in patients with essential hypertension and type 2 diabetes mellitus (T2DM) in Hong Kong, Taiwan, and Thailand. This was a prospective, multicenter, single-arm, open-label study with patients aged 18–75 years with T2DM and essential hypertension and on stable treatment for T2DM. Patients with uncontrolled hypertension were treated with AZL-M 40 mg daily, with the option to uptitrate to 80 mg at 6 weeks. In all, 380 of the 478 patients screened in Hong Kong, Taiwan, and Thailand were enrolled. At week 6, 97 patients (25.5%) were titrated up to AZL-M 80 mg based on BP readings. At 12 weeks, 54.8% of patients reached the blood pressure (BP) goal of <140/85 mm Hg by trough sitting clinic BP (primary endpoint), and 62.8% and 27.0% achieved a BP of <140/90 mm Hg and <130/80 mm Hg, respectively. The efficacy of AZL-M over 12 weeks was also seen in different age and body mass index groups. The incidence of treatment emergent adverse events (TEAEs) was 12.9% before 6 weeks and 16.1% after 6 weeks, and they were mostly mild in severity. The most frequent TEAE was dizziness (4.7%). The incidence of TEAEs leading to study drug discontinuation (4.5%) and drug-related TEAEs (5.0% before 6 weeks; 3.9% after 6 weeks) was low. In patients with essential hypertension and T2DM in Asia, treatment with AZL-M indicated a favorable efficacy and safety profile in achieving target BP.

## 1. Introduction

Hypertension (defined by the World Health Organization (WHO) as systolic blood pressure (SBP) and/or diastolic BP (DBP) ≥140/90 mmHg) is the leading risk factor for cardiovascular disease (CVD). Almost one-third of all deaths globally are attributed to CVD, making CVD the single largest cause of mortality. Complications from hypertension are responsible for 9.4 million of the approximate 17 million CVD-related deaths; this includes 45% of deaths due to heart disease and 51% due to stroke [1].

In Asian countries, the prevalence of hypertension ranges from approximately 11% to 48% [2–5], and in contrast to the observed decline in mean SBP from 1980 to 2008 in Australia, North America, and Western Europe, increases in mean SBP have been observed in South and South East Asia during the same time period [6]. Additionally, recent guidelines published by the American College of Cardiology (ACC) and American Heart Association (AHA) have lowered the definition of high BP to 130/80 mm Hg, thus suggesting a higher burden of illness associated with hypertension [7].

In patients with type 2 diabetes mellitus (T2DM), the prevalence of hypertension is >50%, with many studies reporting a prevalence >75% [8]. In patients with T2DM, comorbid hypertension increases the risk of all-cause mortality by 72% and cardiovascular events by 57% [9], highlighting the importance of controlling BP in this population. The risk for coronary heart disease, left ventricular hypertrophy, congestive heart failure, and stroke is much higher in patients with hypertension and T2DM than with either condition alone [10].

Despite the availability of a variety of antihypertensive medications, BP often remains uncontrolled. In the NHANES study in the United States, the prevalence of “treated” hypertension in adults was approximately 20%, compared to approximately 13% of adults with “controlled” hypertension, which indicated that approximately 7% of the adults were failing to attain a BP < 140/90 mm Hg despite treatment, highlighting the need for newer treatments with superior efficacy and tolerability profiles [11].

The renin-angiotensin-aldosterone-system (RAAS) has been implicated in the association between hypertension and T2DM [12,13], and most treatment guidelines recommend the use of an angiotensin-converting enzyme (ACE) inhibitor or an angiotensin II type 1 receptor blocker (ARB) in these patients, especially in the presence of proteinuria or microalbuminuria [13,14]. Azilsartan medoxomil (AZL-M) is an ARB with long-lasting antihypertensive activity and high selectivity for the angiotensin II type I (AT1) blocker compared to other ARBs [15]. AZL-M is a prodrug, with the active moiety being azilsartan.

The efficacy and safety of AZL-M treatment in hypertension at doses of 40 mg and 80 mg once daily (QD) have been demonstrated in multiple studies conducted in the United States, Latin America, Europe, and Korea [16–19]. AZL-M appears to be more efficacious than valsartan, olmesartan, and candesartan, with a comparable safety and tolerability profile [20]. Data for AZL-M in the Asian population are limited. In a recent phase 3 study in South Korea, AZL-M 40 mg and 80 mg QD reduced SBP significantly more than placebo over 6 weeks [19]. Additionally, a phase 3 randomized controlled trial comparing AZL-M and valsartan has been recently completed in China [21].

Since there are limited data describing the efficacy and safety of AZL-M across different Asian populations, this phase 4 study evaluated AZL-M in Asian adult patients with essential hypertension and T2DM in Hong Kong, Taiwan, and Thailand.

## 2. Materials and Methods

This prospective, multicenter, multicountry, single-arm, open-label phase 4 study was conducted across 34 sites in 3 Asian countries (Hong Kong, Taiwan, and Thailand), from July 2015 to November 2016.

All adult patients (aged 18–75 years) with established diagnosis of uncontrolled essential hypertension and T2DM were eligible for the study. Uncontrolled hypertension was defined as SBP ≥140 mm Hg to <180 mm Hg or DBP ≥85 mm Hg and <110 mm Hg at study entry.

To be eligible, T2DM was required to be treated by stable lifestyle intervention or by oral antidiabetic drugs that were stable, including no dose adjustment within 12 weeks before baseline, with hemoglobin A1c (HbA1c) < 9.5% at study entry.

Key exclusion criteria included the following: uncontrolled essential hypertension despite concurrent treatment with 3 antihypertensive medications from different classes; type 1 or poorly controlled T2DM (HbA1c ≥ 9.5%); congestive heart failure; clinically relevant cardiac arrhythmias; severe obstructive coronary artery disease; severe renal impairment; and hyperkalemia (serum potassium >5.0 mEq/L).

All eligible patients were treated with AZL-M at a starting dose of 40 mg QD. For patients currently receiving antihypertensive medications, they were either switched to AZL-M (“switched” group) or received AZL-M in addition to their ongoing treatment (“add-on”group). The “switched” group means patients that received ACEI or ARB at baseline, and the “add-on” group means patients received other antihypertensive drugs than ACEI/ARB. If the BP goal (<140/85 mm Hg) was not reached after 6 weeks of treatment, the dose of AZL-M was uptitrated to 80 mg QD. Antidiabetic treatment was to remain stable for the duration of the treatment period (12 weeks).

Concomitant treatment with lithium, insulin, spironolactone, aliskiren, ACE inhibitors, and other ARBs was prohibited during the study.

BP measurement machine was calibrated in each center and measured by the same nurse in each center. The adverse events were done by an interview during site visit.

This study was registered at ClinicalTrials.gov (NCT02517866; https://clinicaltrials.gov/ct2/show/NCT02517866) on August 7, 2015, and was conducted in accordance with the protocol, the ethical principles that have their origin in the Declaration of Helsinki, the ICH E6 GCP guidance, and all applicable regulations. The study was reviewed and approved by the local or central IRBs/IECs of all study sites. Each subject (or the subject's legally authorized representative) signed and dated the informed consent form before undergoing any study participation.

The primary objective of the study was to determine the percentage of patients reaching a BP goal of <140/85 mm Hg (SBP <140 mm Hg and DBP <85 mm Hg) by trough sitting clinic BP at week 12. The sitting clinic BP was measured in triplicate and averaged at each study visit using a validated device. The BP goal of 140/85 mm Hg was based on the recommendations of the European Society of Hypertension-European Society of Cardiology (ESH-ESC) 2013 treatment guidelines for patients with hypertension and diabetes [10,14].

Secondary objectives included the following: to determine the proportion of patients reaching other BP goals after 12 weeks of treatment with AZL-M (BP < 140/90 mm Hg, BP < 130/80 mm Hg, SBP <140 mm Hg, DBP <85 mm Hg, DBP <90 mm Hg, SBP <130 mm Hg, or DBP <80 mm Hg); to determine the proportion of patients reaching BP goals (<140/85 mm Hg or <130/80 mm Hg) after treatment with AZL-M according to their baseline treatment status (i.e., treatment-naïve, switched to AZL-M, or AZL-M added to previous treatment); to determine the change from baseline till 12 weeks in SBP and DBP; and to assess the safety and tolerability of AZL-M.

Additional objectives were to determine the proportion of patients reaching BP goals after 6 weeks of treatment with AZL-M (BP < 140/85 mm Hg, BP < 140/90 mm Hg, BP < 130/80 mm Hg, SBP <140 mm Hg, DBP <85 mm Hg, DBP <90 mm Hg, SBP <130 mm Hg, or DBP <80 mm Hg); to determine the change from baseline till 6 weeks in SBP and DBP; and to assess change in HbA1c from baseline at week 12, excluding patients who were on an RAAS inhibitor within 3 months before baseline. The proportion of patients meeting the endpoints described above was estimated, i.e., calculated after adjusting for relevant factors as described in the statistical analysis.

Statistical analysis was performed using the SAS System, Version 9.4, on a Windows platform. The full analysis set (FAS), which consisted of all enrolled patients who took at least one dose of AZL-M, was the primary dataset for efficacy analyses. The safety analysis set, which consisted of all patients who took at least 1 dose of AZL-M, was used for demographic and baseline characteristic summaries and routine safety analysis.

The primary endpoint, which was the percentage of patients with BP < 140/85 mm Hg at week 12, was determined using a generalized estimated equation (GEE) logistic regression model, including SBP as the covariate, country, baseline hypertension treatment (BHT) status, visit (week 6 and week 12), and BHT and visit interaction as fixed factors. Unless otherwise specified, all statistical inferences used a 2-sided 0.05 significance level.

Subgroup analyses were performed for age (<65 and ≥ 65 years) and baseline body mass 148 index (BMI; <23 kg/m^2^ and ≥23 kg/m^2^; <30 kg/m^2^ and ≥30 kg/m^2^).

Secondary and exploratory endpoints were evaluated similarly to the analysis performed for the primary endpoint. Mean change from baseline in trough SBP (or DBP) was estimated by visit (week 6 and week 12) using the analysis of covariance (ANCOVA) model with fixed effects of country and BHT; baseline SBP (or DBP) was included as a covariate.

A sample size of 290 patients was considered sufficient to reach the primary objective with ±7.5% as the width of the 99% confidence interval (CI) for the responder rate, assuming that 55% of the patients would meet the BP goal. Assuming a 20% dropout and the recruitment target of individual sites, the total sample size for the study was estimated to be approximately 363.

## 3. Results

### 3.1. Patient Disposition

**Figure 1:**
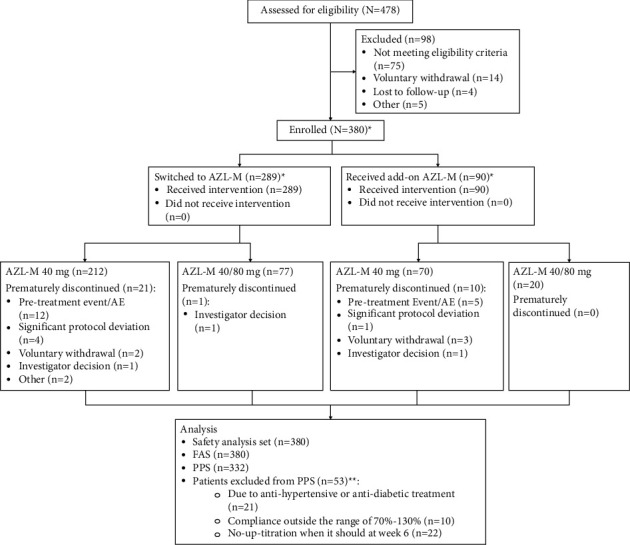
Flow diagram for patients in the study. AE, adverse event; AZL-M, azilsartan. 168, medoxomil; FAS, full analysis set; and PPS, per protocol set. ^∗^One patient from the “treatment-naïve” group was considered only in the overall analysis. ^∗∗^The same patient meeting multiple per protocol set exclusion criteria is counted under each criterion.

A total of 478 patients were screened for the study, of which 380 were enrolled; 289 patients were switched to AZL-M, 90 received AZL-M in addition to their ongoing treatment, and one patient was treatment-naïve (see Figure 1).Of these, 348 patients (91.6%) completed 12 weeks of treatment with AZL-M and 354 (93.2%) completed all planned study visits. Of the 380 enrolled patients, 139 were in Taiwan (15 sites), 219 in Thailand (17 sites), and 22 in Hong Kong (2 sites).

### 3.2. Patient Demographics and Baseline Characteristics

The key patient demographics and baseline characteristics are shown in Table 1. At baseline, mean patient age was 61.6 years (58.2% were aged <65 years). Mean BMI was 27.6 kg/m^2^, and mean HbA1c level was 7.00%. Slightly more than half of the patients (53.2%) were previously treated with ACE inhibitors or ARBs for hypertension. There were no clinically meaningful differences between BHT status groups for sitting clinic SBP or DBP or HbA1c.

### 3.3. Treatment Exposure

Overall, the mean exposure of patients to AZL-M was 80.2 days, and most patients (97.4%) were 70% to 130% compliant. The treatment duration was comparable between BHT status groups.

### 3.4. Efficacy

The results from the analyses of the primary efficacy endpoint, percentage of patients with trough BP < 140/85 mm Hg, are presented in Table 2. After 12 weeks of treatment, 61% of the patients achieved trough BP < 140/85 mm Hg. Using the GEE logistic regression model (adjusted for baseline BP, country, and visit), the estimated percentage of patients meeting the target BP was 54.8% (95% confidence interval (CI) 47.75, 61.70). The estimated proportions of patients meeting the BP target goal of <140/85 mm Hg for the switched and add-on groups were 53.4% and 61.0%, respectively, with overlapping 95% CIs. The estimated proportion of patients reaching the BP goal of <140/85 mm Hg at 6 weeks was 58.7% (95% CI 51.75, 65.37).

The results from the PPS analysis were consistent, with 59.0% (95% CI 51.57, 66.00) of the patients reaching the target trough BP level of 140/85 mm Hg at 12 weeks.

At week 6, 119 patients (31.3%) had not achieved BP target <140/85 mm Hg and were eligible for uptitration to AZL-M 80 mg QD. Twenty-two of these patients were not uptitrated as decided by the investigator, mainly due to safety reasons; this included 14 patients in the switched group and 8 patients in the add-on group. Of the 97 patients (25.5% of 380) who were uptitrated, the estimated proportion (using the GEE regression model) of patients who 212 achieved target BP < 140/85 mm Hg at 12 weeks was 15.4% (95% CI 8.69, 25.87).

The results for BP < 140/85 mm Hg by BHT groups are shown in Table 3.

The estimated proportion (using GEE regression models) of patients meeting target BP at 12 weeks was numerically higher for patients who were treated with thiazides before baseline (83.8%; 95% CI 55.08, 95.64) than for those who were treated with calcium channel blockers (CCBs) (52.6%; 95% CI 42.76, 62.21) or ACE inhibitors/ARBs (57.3%; 95% CI 47.73, 66.37). Note that the proportions for the thiazides group were based on data from only 29 patients, resulting in wide confidence intervals. Most of the patients across these 3 treatment groups were switched to AZL-M at baseline 84% of those in the CCB group, 98% of those in the ACE inhibitors/ARBs group, and 100% of those in the thiazides group.

Table 2 also shows the results for the additional BP goals of <140/90 mm Hg and <130/80 mm Hg. At 12 weeks, the estimated proportion of patients achieving a BP of <130/80 mm Hg and <140/90 mm Hg was 27% and 63%, respectively. While a greater proportion of patients achieved the BP goal of 130/80 mm Hg at 12 versus 6 weeks (27.0% [95% CI 20.06, 35.18] vs. 22.9% [95% CI 16.60, 30.67]), the converse was observed for patients who achieved the BP goal of 140/90 mm Hg (62.8% at 12 weeks [95% CI 56.06, 69.11] vs. 63.8% at 6 weeks [95% CI 57.26, 69.93]).

**Figure 2:**
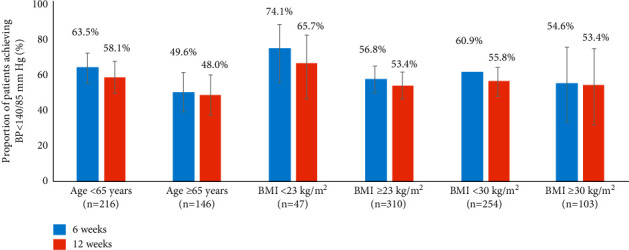
Percentage of patients with trough BP < 140/85 mm Hg by age and BMI subgroup analysis using GEE (logistic regression analysis): FAS and LOCF and number of patients with nonmissing values and used as denominator to calculate percentage. The percentages and 95% confidence intervals are shown. BMI, body mass index; FAS, full analysis set; GEE, generalized estimated equation; and LOCF, last observation carried forward.

The efficacy of AZL-M in achieving the BP goal of <140/85 mm Hg over 12 weeks was also observed across all age and BMI groups (see Figure 2).

Changes in BP from baseline are shown in Supplementary Figure 1. At 12 weeks, a mean reduction of 14.1 mm Hg in SBP and 5.4 mm Hg in DBP was observed. For both SBP and DBP, mean reductions were numerically larger at 12 weeks compared to 6 weeks.

No clinically meaningful changes were seen in HbA1c levels from baseline to week 12 (mean change 0.16%; 95% CI 0.057, 0.266).

### 3.5. Safety

The treatment was well tolerated (Table 4). Overall, 26.3% of the patients experienced at least 1 treatment emergent adverse event (TEAE). The incidence of TEAEs was broadly similar in the AZL-M 40 mg group before and after week 6 (12.9% vs. 15.5%) and across patients taking AZL-M 40 mg and 80 mg after week 6 (15.5% vs. 17.5%).

The list of AEs reported by ≥ 0.5% of all patients is provided in Supplementary Table 1. The most frequently reported TEAEs (reported by ≥ 1% of all patients) over 12 weeks were dizziness (4.7%), upper respiratory tract infection (2.9%), headache (2.4%), hyperkalemia (2.1%), nasopharyngitis (1.6%), diarrhea (1.3%), fatigue (1.1%), hypoglycemia (1.1%), and hypotension (1.1%).

A total of 32 patients (8.4%) experienced treatment-related AEs (TRAEs) over 12 weeks, as shown in Supplementary Table 1. The most frequently reported TRAEs (reported by ≥ 1% of all patients) were dizziness (2.6%) and hyperkalemia (1.6%).

TEAEs leading to study drug discontinuation occurred in 4.5% of all patients over 12 weeks; dizziness (1.6%; n = 6) was the most frequently reported AE leading to discontinuation, followed by hypotension (0.8%; n = 3). No patient discontinued AZL-M due to TEAEs after being titrated up to 80 mg.

Most TEAEs were mild in severity. Ten serious adverse events (SAEs) were reported for 8 patients (2.1%). Acute kidney injury, reported in 3 patients (0.8%), was the most frequently reported SAE. Of the 10 SAEs, 2 (hypotension and acute kidney injury; 1 patient each) were considered related to study drug.

One patient developed respiratory failure on day 33 of treatment and died after day 51 of study enrolment. The patient was a 68-year-old Asian male (“switched” group; AZL-M 40 mg QD) suffering from essential hypertension, T2DM, hypertensive heart disease, hyperlipidemia, embolic cerebral infarction, and chronic gastritis. Following the respiratory failure on day 33, the patient developed cardiac arrest, heart failure, and hypoxic encephalopathy. The probable cause of death was respiratory failure, and the investigator considered the event to be unrelated to AZL-M.

The change in vital signs (pulse rate and body temperature) and weight over the study duration was small and considered not clinically meaningful.

## 4. Discussion

This was the first phase 4 study to evaluate AZL-M in adult patients with essential hypertension and T2DM in Hong Kong, Taiwan, and Thailand. Outcomes were assessed across multiple subgroups at 2 time points. Over 90% of the patients completed 12 weeks of treatment with AZL-M.

In this study, switching to or adding AZL-M to current antihypertensive therapy in patients with hypertension and T2DM resulted in significant improvement in BP control over 12 weeks, as measured by trough clinic measurements. At 12 weeks, 54.8% of patients achieved BP < 140/85 mm Hg, a target recommended by the ESH-ESC guidelines for patients with hypertension and diabetes [14]. Nearly 2 in 3 patients achieved the more “standard” goal of BP < 140/90 mm Hg. Some guidelines, e.g., the ACC/AHA guidelines [7], the Canadian Hypertension Education Program, and the International Diabetes Federation, recommend a lower BP target of 130/80 mm Hg in those with T2DM [10]; in our study, 27.0% of patients reached that goal. This BP reduction was achieved in a population in which BP was uncontrolled despite receiving stable ongoing antihypertensive treatment (only 1 patient was “treatment-naïve”), and that significant BP reduction was seen in patients who switched to AZL-M as well as those receiving it as an add-on to their current treatment.

A slightly higher (but statistically insignificant) percentage of patients who added AZL-M to their current treatment achieved the BP target of 140/85 mm Hg compared to those who switched (61.0% vs. 53.4%). While this may simply reflect incremental efficacy due to addition of an antihypertensive drug rather than substitution, this finding warrants further investigation.

Interestingly, the efficacy in terms of achieving BP < 140/85 mm Hg appeared to be the highest (83.8%) in the group of patients who received thiazides prior to baseline, all of whom switched to AZL-M during the study. This finding suggests that ACE inhibitors/ARBs should be considered in this population [13]. However, the current findings should be interpreted with caution due to the small number of patients (*n* = 30) in the thiazide group who were switched to treatment with AZL-M.

Due to nonresponse at 6 weeks, approximately 1 in 4 patients were uptitrated from 40 mg to 80 mg QD of AZL-M. The higher dose of AZL-M was effective in achieving target BP in about 15% of these patients, suggesting that uptitration should be considered in this relatively “treatment-resistant” group.

For the BP goals of 140/85 mm Hg and 140/90 mm Hg, slightly fewer patients achieved the BP goals at 12 weeks compared to 6 weeks. This may indicate a slight decline in treatment effect over time. However, this is more likely to be a chance occurrence in view of the following: the small magnitude of difference across the 2 time points; overlap of the 95% confidence intervals at 6 and 12 weeks; and the reversal of findings for a BP goal of 130/80 mm Hg (slightly more patients achieved the goal at 12 weeks compared to 6 weeks); and for change in SBP and DBP (numerically larger decline from baseline at 12 weeks than at 6 weeks). Frequent BP assessments over a longer period would be more suitable to identify secular trends, if any.

The efficacy of AZL-M was observed across all age and BMI subgroups in this study. The BMI levels in the current study were lower than those observed in previous studies with AZL-*M* (in non-Asian countries) conducted in patients with prediabetes mellitus and T2DM [22]. This is consistent with the literature showing that T2DM patients have a lower BMI in East Asian countries [23].

Results from our study were broadly comparable to those reported in previous clinical studies with AZL-M. In previous studies, response based on joint reduction in SBP and DBP was defined as SBP <140 mm Hg and/or reduction from baseline of ≥20 mm Hg and DBP <90 mm Hg and/or reduction from baseline of ≥10 mm Hg [16,19,24–26]. Summary results from the studies are shown in Supplementary Table 2.

Treatment with AZL-M 40 mg and 80 mg QD over 12 weeks in patients with hypertension and T2DM was well tolerated. The incidence of adverse events was similar in the 40 mg and 80 mg groups and was similar before and after 6 weeks of treatment. Dizziness was the most frequently reported TEAE (4.7%). The TRAEs were primarily expected events related to underlying hypertension as well as those known to be associated with RAAS blocking agents [16,17,19]. Dizziness was also the most frequent event leading to discontinuation. A single death was reported, which was considered to be unrelated to the study drug. The safety and tolerability profile of AZL-M was consistent with the results from clinical studies conducted in other racial/ethnic groups [16,17,19].

A key limitation of our study was that it was conducted as a single-arm uncontrolled study, thus limiting the conclusions that could be drawn regarding the efficacy of AZL-M. However, since the efficacy and safety of AZL-M have previously been demonstrated in placebo-controlled studies in adult patients with essential hypertension in United States, Latin America, Europe, and Korea [16–19], this study design was appropriate for achieving the objective of generating relevant data in Hong Kong, Taiwan, and Thailand. The study was not powered to detect significant changes in different subgroups. Therefore, the results for the subgroups cannot be considered conclusive and deserve further investigation in a larger patient series.

Future studies should focus on better understanding the subgroups which demonstrated the greatest efficacy with AZL-M, e.g., those patients whose BP was uncontrolled on thiazides. Differences in efficacy based on whether AZL-M is added on to or switched with the ongoing treatment deserve further investigation. Generating long-term (i.e., beyond 12 weeks treatment duration) outcome data, including cardiovascular events, quality of life, and healthcare resource utilisation, in patients from Asian countries is also of interest.

## 5. Conclusions

In patients with essential hypertension and T2DM in Asia, treatment with AZL-M indicated a favorable efficacy and safety profile in achieving target BP. The safety and tolerability profile of AZL-M in Asian patients was consistent with the known profile of AZL-M.

## Figures and Tables

**Table 1:** Key demographic and baseline characteristics (safety analysis set).

Parameter	Number of patients by BHT status groups (%)		
	Switched	Add-on	Overall
*N*	289	90	380
Female, *n* (%)	155 (53.6)	42 (46.7)	197 (51.8)
Age, years, mean (SD)	61.3 (9.95)	62.5 (9.17)	61.6 (9.77)
Age category, years, *n* (%)			
≥65 to <75	116 (40.1)	43 (47.8)	159 (41.8)
<65	173 (59.9)	47 (52.2)	221 (58.2)
≥45 to <65	151 (52.2)	42 (46.7)	194 (51.1)
<45	22 (7.6)	5 (5.6)	27 (7.1)
Body weight, kg, mean (SD)	72.0 (15.71)	72.1 (13.05)	72.0 (15.07)
BMI, kg/m^2^, mean (SD)	27.6 (4.51)	27.8 (3.85)	27.6 (4.35)
BMI category, kg/m^2^, *n* (%)			
<23	39 (13.5)	10 (11.1)	49 (12.9)
≥23	244 (84.4)	80 (88.9)	325 (85.5)
<30	202 (69.9)	65 (72.2)	268 (70.5)
≥30	81 (28.0)	25 (27.8)	106 (27.9)
HbA1c, mmoL/moL, mean (SD)	7.00 (0.901)	7.01 (0.796)	7.00 (0.875)
Baseline antihypertensive treatment, *n* (%)^∗^			
ACE inhibitor or ARB	198 (68.5)	4 (4.4)	202 (53.2)
CCB	94 (32.5)	18 (20.0)	112 (29.5)
Thiazide	30 (10.4)	0	30 (7.9)
Other	52 (18.0)	68 (75.6)	121 (31.8)
Clinical sitting SBP, mm Hg, mean (SD)	152.2 (11.58)	152.7 (12.08)	152.3 (11.68)
Clinical sitting DBP, mm Hg, mean (SD)	84.8 (9.87)	84.0 (8.94)	84.6 (9.65)

ACE, angiotensin-converting enzyme; ARB, angiotensin receptor blocker; BHT, baseline hypertension treatment; BMI, body mass index; CCB, calcium channel blocker; DBP, diastolic blood pressure; HbA1c, glycosylated hemoglobin; SBP, systolic blood pressure; SD, standard deviation. One patient from the treatment-naïve group was considered only in the overall analysis. ^∗^The sum of all treatments exceeds 380 as a single patient may have been receiving up to 2 antihypertensive treatments at baseline.

**Table 2:** Percentage of patients with trough BP < 140/85 mm Hg, BP < 130/80 mm Hg, and BP 198 < 140/90 mm Hg using GEE (logistic regression model): FAS and LOCF.

Trough BP	Visit	Switched (*n* = 289)	Patients	
			Add-on (*n* = 90)	Overall (*N* = 380)
<140/85 mm Hg	Week 6			
	*N*′	276	85	362
	*n* (%)	178 (64.5)	54 (63.5)	233 (64.4)
	Adjusted %^*∗*^	58.9	58.2	58.7
	99% CI	(48.99, 68.12)	(41.22, 73.48)	(49.53, 67.36)
	95% CI	(51.38, 66.01)	(45.25, 70.16)	(51.75, 65.37)
	Week 12			
	*N*′	276	85	362
	*n* (%)	165 (59.8)	56 (65.9)	221 (61.0)
	Adjusted %^∗^	53.4	61.0	54.8
	99% CI	(43.40, 63.08)	(44.07, 75.70)	(45.54, 63.78)
	95% CI	(45.76, 60.82)	(48.15, 72.56)	(47.75, 61.70)

<130/80 mm Hg	Week 6			
	*N*′	276	85	362
	*n* (%)	83 (30.1)	28 (32.9)	111 (30.7)
	Adjusted %^∗^	22.6	24.9	22.9
	99% CI	(14.46, 33.44)	(13.09, 42.15)	(14.93, 33.40)
	95% CI	(16.15, 30.61)	(15.39, 37.64)	(16.60, 30.67)
	Week 12			
	*N*′	276	85	362
	*n* (%)	93 (33.7)	34 (40.0)	127 (35.1)
	Adjusted %^∗^	25.9	31.8	27.0
	99% CI	(17.01, 37.26)	(18.66, 48.66)	(18.18, 37.99)
	95% CI	(18.88, 34.34)	(21.37, 44.45)	(20.06, 35.18)

<140/90 mm Hg	Week 6			
	*N*′	276	85	362
	*n* (%)	186 (67.4)	56 (65.9)	243 (67.1)
	Adjusted %^∗^	64.0	63.0	63.8
	99% CI	(54.59, 72.41)	(45.37, 77.74)	(55.12, 71.72)
	95% CI	(56.89, 70.51)	(49.65, 74.63)	(57.26, 69.93)
	Week 12			
	*N*′	276	85	362
	*n* (%)	182 (65.9)	58 (68.2)	240 (66.3)
	Adjusted %^∗^	62.2	65.9	62.8
	99% CI	(52.51, 71.04)	(48.42, 79.92)	(53.88, 70.96)
	95% CI	(54.88, 69.04)	(52.74, 77.00)	(56.06, 69.11)

CI, confidence interval; FAS, full analysis set; GEE, generalized estimated equation; LOCF, last observation carried forward. *N*' = number of patients with nonmissing values and used as denominator to calculate percentage. ^*∗*^The GEE logistic regression model was adjusted for baseline SBP (systolic blood pressure), country, baseline hypertension treatment status, and visit. One patient from the treatment-naïve group was considered only in the overall analysis.

**Table 3:** Percentage of patients treated with CCBs, ACE/ARBs, and thiazides before baseline 215 reaching BP < 140/85 mm Hg using GEE (logistic regression model): FAS and LOCF.

Visit	Patients with trough BP < 140/85 mm Hg		
	CCBs	ACE inhibitors/ARBs	Thiazides
	(*N* = 112)	(*N* = 202)	(*N* = 30)
Week 6			
*N*′	109	193	29
*n* (%)	63 (57.8)	123 (63.7)	23 (79.3)
Adjusted %^∗^	58.8	57.3	72.9
99% CI	(45.36, 71.12)	(44.83, 68.92)	(45.97, 89.46)
95% CI	(48.60, 68.38)	(47.81, 66.30)	(52.83, 86.57)
Week 12			
*N*′	109	193	29
*n* (%)	57 (52.3)	123 (63.7)	25 (86.2)
Adjusted %^∗^	52.6	57.3	83.8
99% CI	(39.75, 65.08)	(44.73, 69.01)	(43.81, 97.18)
95% CI	(42.76, 62.21)	(47.73, 66.37)	(55.08, 95.64)

ACE, angiotensin-converting enzyme; ARB, angiotensin receptor blocker; CCB, calcium channel blocker; CI, confidence interval; FAS, full analysis set; GEE, generalized estimated equation; LOCF, last observation carried forward. *N*' = number of patients with nonmissing values and used as denominator to calculate percentage. ^∗^The GEE logistic regression model was adjusted for baseline SBP (systolic blood pressure), country, baseline hypertension treatment status, and visit. One patient from the treatment-naïve group was considered only in the overall analysis.

**Table 4:** Frequency of AEs : safety analysis set and overall population.

AEs	Overall (*N* = 380)									
	Before week 6 *n* = 380				After week 6 *n* = 355				Total (*N* = 380)	
	AZL-M 40 mg (*n* = 380)		AZL-M 40 mg (*n* = 258)		AZL-M 80 mg (*n* = 97)		Total (*n* = 355)					
	Events n	Patients, *n* (%)	Events n	Patients, *n* (%)	Events n	Patients, *n* (%)	Events n	Patients, *n* (%)	Events n	Patients, *n* (%)
TEAEs	78	49 (12.9)	70	40 (15.5)	29	17 (17.5)	99	57 (16.1)	193	100 (26.3)
Related to AZL-M	23	19 (5)	15	12 (4.7)	2	2 (2.1)	17	14 (3.9)	41	32 (8.4)
Not related to AZL-M	55	37 (9.7)	55	30 (11.6)	27	15 (15.5)	82	45 (12.7)	152	79 (20.8)
Related to study procedures	1	1 (0.3)	1	1 (0.4)	0	0	1	1 (0.3)	2	2 (0.5)
Not related to study procedures	77	48 (12.6)	69	39 (15.1)	29	17 (17.5)	98	56 (15.8)	191	98 (25.8)
Leading to study drug discontinuation	14	14 (3.7)	4	3 (1.2)	0	0	4	3 (0.8)	18	17 (4.5)
Mild	70	44 (11.6)	60	32 (12.4)	20	15 (15.5)	80	47 (13.2)	164	86 (22.6)
Moderate	3	3 (0.8)	8	6 (2.3)	6	4 (4.1)	14	10 (2.8)	19	13 (3.4)
Severe	5	5 (1.3)	2	2 (0.8)	3	1 (1)	5	3 (0.8)	10	8 (2.1)
Serious TEAEs	4	4 (1.1)	3	3 (1.2)	3	1 (1)	6	4 (1.1)	10	8 (2.1)
Deaths	1	1 (0.3)	0	0	0	0	0	0	1	1 (0.3)

270 AE, adverse event; AZL-M, azilsartan medoxomil; TEAE, treatment-related adverse event.

## Data Availability

The data presented in this manuscript will not be shared due to patient privacy and commercial confidentiality.

## Supplementary Materials

Supplementary Materials
The supplementary materials consist of three files, two tables (Supplementary Table 1, listing the common adverse events reported in the study, and Supplementary Table 2, summarizing results from previous studies conducted with AZL-M) and one figure (Supplementary Figure 1, displaying the change from baseline in trough sitting SBP and DBP in mm Hg). All files have been submitted in MS Word format.

## References

[1] World Health Organisation (2013). A global brief on hypertension. http://ish-world.com/data/uploads/global_brief_hypertension.pdf.

[2] World Health Organisation (2018). Cardiovascular diseases (CVDs). http://www.who.int/mediacentre/factsheets/fs317/en/.

[3] Neupane D., McLachlan C. S., Sharma R. (2014). Prevalence of hypertension in member countries of South asian association for regional cooperation (SAARC). *Medicine (Baltimore)*.

[4] Kong Department of Health H. (2018). Hypertension is preventable and treatable”. http://www.chp.gov.hk/en/view_content/28603.html.

[5] Aekplakorn W., Sangthong R., Kessomboon P. (2012). Changes in prevalence, awareness, treatment and control of hypertension in Thai population, 2004-2009. *Journal of Hypertension*.

[6] Bromfield S., Muntner P. (2013). High blood pressure: the leading global burden of disease risk factor and the need for worldwide prevention programs. *Current Hypertension Reports*.

[7] Whelton P. K., Carey R. M., Aronow W. S., Casey D. E., Collins K. J., Himmelfarb C. D. (2018). 2017, ACC/AHA/aapa/ABC/ACPM/AGS/APhA/ASH/ASPC/NMA/pcna guideline for the prevention, detection, evaluation, and management of high blood pressure in adults: a report of the American College of Cardiology/American heart association task force on clinical practice guidelines. *Journal of the American College of Cardiology*.

[8] Colosia A., Khan S., Palencia R. (2013). Prevalence of hypertension and obesity in patients with type 2 diabetes mellitus in observational studies: a systematic literature review. *Diabetes, Metabolic Syndrome and Obesity: Targets and Therapy*.

[9] Chen G., McAlister F. A., Walker R. L., Hemmelgarn B. R., Campbell N. R. C. (2011). Cardiovascular outcomes in framingham participants with diabetes. *Hypertension*.

[10] Grossman A., Grossman E. (2017). Blood pressure control in type 2 diabetic patients. *Cardiovascular Diabetology*.

[11] Wright J. D., Hughes J. P., Ostchega Y., Yoon S. S., Nwankwo T. (2011). Mean systolic and diastolic blood pressure in adults aged 18 and over in the United States, 2001-2008. *Natl Health Stat Report*.

[12] Lastra G., Syed S., Kurukulasuriya L. R., Manrique C., Sowers J. R. (2014). Type 2 diabetes mellitus and hypertension. *Endocrinology and Metabolism Clinics of North America*.

[13] Cryer M. J., Horani T., DiPette D. J. (2016). Diabetes and hypertension: a comparative review of current guidelines. *Journal of Clinical Hypertension*.

[14] Mancia G., Fagard R., Narkiewicz K. (2013). Task Force Members: 2013 ESH/ESC Guidelines for the management of arterial hypertension. *Journal of Hypertension*.

[15] Angeloni E. (2016). Azilsartan medoxomil in the management of hypertension: an evidence-based review of its place in therapy. *Core Evidence*.

[16] Bakris G. L., Sica D., Weber M. (2011). The comparative effects of azilsartan medoxomil and olmesartan on ambulatory and clinic blood pressure. *Journal of Clinical Hypertension*.

[17] Sica D., White W. B., Weber M. A. (2011). Comparison of the novel angiotensin II receptor blocker azilsartan medoxomil vs valsartan by ambulatory blood pressure monitoring. *Journal of Clinical Hypertension*.

[18] Bramlage P., Schmieder R. E., Schmieder R. E. (2015). The renin-angiotensin receptor blocker azilsartan medoxomil compared with the angiotensin-converting enzyme inhibitor ramipril in clinical trials versus routine practice: insights from the prospective EARLY registry. *Trials*.

[19] Juhasz A., Wu J., Hisada M., Tsukada T., Jeong M. H. (2018). Efficacy and safety of azilsartan medoxomil, an angiotensin receptor blocker, in Korean patients with essential hypertension. *Clinical Hypertension*.

[20] De Caterina A., Harper A. R., Cuculi F. (2012). Critical evaluation of the efficacy and tolerability of azilsartan. *Vascular Health and Risk Management*.

[21] Nct02480764 (2018). TAK-491 (azilsartan medoxomil) compared to valsartan in Chinese participants with hypertension. https://clinicaltrials.gov/ct2/show/NCT02480764.

[22] White W. B., Cuadra R. H., Lloyd E., Bakris G. L., Kupfer S. (2016). Effects of azilsartan medoxomil compared with olmesartan and valsartan on ambulatory and clinic blood pressure in patients with type 2 diabetes and prediabetes. *Journal of Hypertension*.

[23] Ma R. C. W., Chan J. C. N. (2013). Type 2 diabetes in East Asians: similarities and differences with populations in Europe and the United States. *Annals of the New York Academy of Sciences*.

[24] Nct00696241 (2018). Efficacy and safety of azilsartan medoxomil in participants with essential hypertension. https://clinicaltrials.gov/ct2/show/results/NCT00696241.

[25] White W. B., Weber M. A., Sica D. (2011). Effects of the angiotensin receptor blocker azilsartan medoxomil versus olmesartan and valsartan on ambulatory and clinic blood pressure in patients with stages 1 and 2 hypertension. *Hypertension*.

[26] Nct00696436 (2018). An efficacy and safety study of azilsartan medoxomil compared to valsartan and olmesartan in participants with essential hypertension. https://clinicaltrials.gov/ct2/show/study/NCT00696436.

