# Supplementary material for: A Prospective Study of Azilsartan Medoxomil in the Treatment of Patients with Essential Hypertension and Type 2 Diabetes in Asia
Source: Int J Hypertens. 2022 Jan 7;2022:2717291. doi: 10.1155/2022/2717291 (PMC8759883; doi:10.1155/2022/2717291)
Supplement: Supplementary Materials — The supplementary materials consist of three files, two tables (Supplementary Table 1, listing the common adverse events reported in the study, and Supplementary Table 2, summarizing results from previous studies conducted with AZL-M) and one figure (Supplementary Figure 1, displaying the change from baseline in trough sitting SBP and DBP in mm Hg). All files have been submitted in MS Word format. [file 2717291.f1.zip › 2717291.f1/Supplementary Figure 1_16th Jan '20.docx]

Supplementary Figure 1. Analysis of change from baseline in trough sitting SBP and DBP (mm Hg) by visit: FAS, LOCF
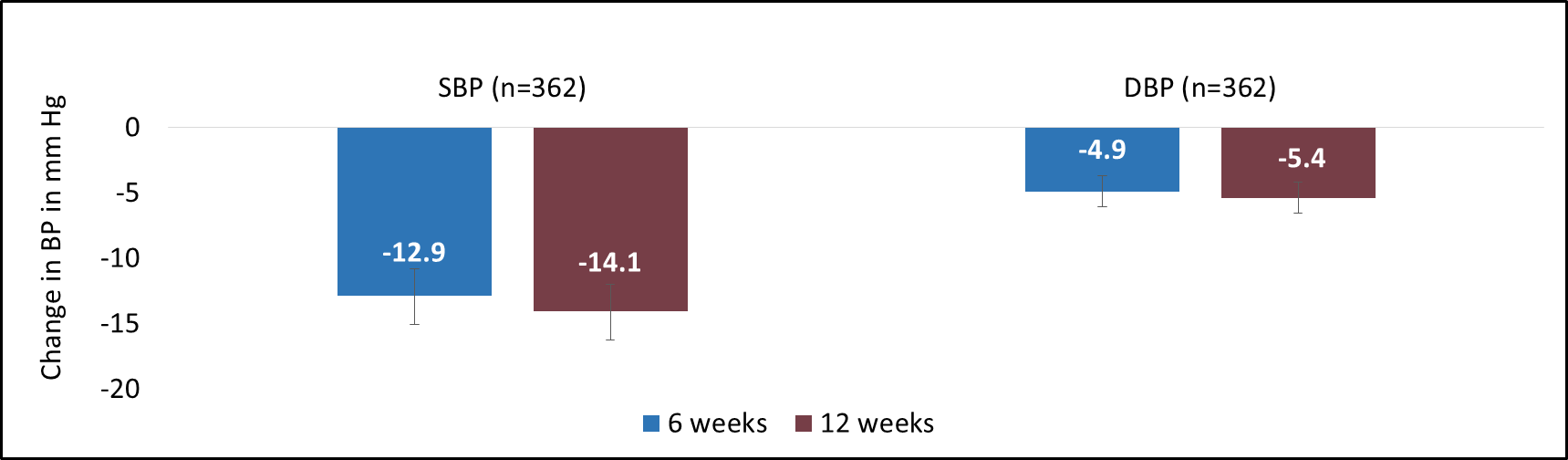


Abbreviations: BP, blood pressure; DBP, diastolic blood pressure; FAS, full analysis set; GEE, generalized estimated equation; LOCF, last observation carried forward; SBP, systolic blood pressure.

Number of patients with non-missing values used as denominator to calculate percentage.

The figure shows the least square mean (standard error) change in BP and 95% confidence intervals.
