# Supplementary material for: A Prospective Study of Azilsartan Medoxomil in the Treatment of Patients with Essential Hypertension and Type 2 Diabetes in Asia
Source: Int J Hypertens. 2022 Jan 7;2022:2717291. doi: 10.1155/2022/2717291 (PMC8759883; doi:10.1155/2022/2717291)
Supplement: Supplementary Materials — The supplementary materials consist of three files, two tables (Supplementary Table 1, listing the common adverse events reported in the study, and Supplementary Table 2, summarizing results from previous studies conducted with AZL-M) and one figure (Supplementary Figure 1, displaying the change from baseline in trough sitting SBP and DBP in mm Hg). All files have been submitted in MS Word format. [file 2717291.f1.zip › 2717291.f1/Supplementary Table 1_16th Jan '20.docx]

Supplementary Table 1. TEAEs and TRAEs (≥0.5% in safety analysis set) by preferred term: Overall population

| **AEs** | **Overall (N=380)** | | | | | | | | | |
| --- | --- | --- | --- | --- | --- | --- | --- | --- | --- | --- |
|  | **Before Week 6**  **n=380** | | **After Week 6**  **n=355** | | | | | | **Total**  **(N=380)** | |
|  | **AZL-M 40 mg**  **(n=380)** | | **AZL-M 40 mg**  **(n=258)** | | **AZL-M 80 mg**  **(n=97)** | | **Total**  **(n=355)** | |  |  |
|  | **n** | **%** | **n** | **%** | **n** | **%** | **n** | **%** | **n** | **%** |
| **Patients with any TEAEs** | **49** | **12.9** | **40** | **15.5** | **17** | **17.5** | **57** | **16.1** | **100** | **26.3** |
| **Dizziness** | 14 | 3.7 | 4 | 1.6 | 1 | 1.0 | 5 | 1.4 | 18 | 4.7 |
| **Upper respiratory tract infection** | 6 | 1.6 | 2 | 0.8 | 2 | 2.1 | 4 | 1.1 | 11 | 2.9 |
| **Headache** | 7 | 1.8 | 2 | 0.8 | 1 | 1.0 | 3 | 0.8 | 9 | 2.4 |
| **Hyperkalemia** | 0 | 0 | 7 | 2.7 | 1 | 1.0 | 8 | 2.3 | 8 | 2.1 |
| **Nasopharyngitis** | 1 | 0.3 | 2 | 0.8 | 1 | 1.0 | 3 | 0.8 | 6 | 1.6 |
| **Diarrhea** | 2 | 0.5 | 2 | 0.8 | 1 | 1.0 | 3 | 0.8 | 5 | 1.3 |
| **Fatigue** | 3 | 0.8 | 1 | 0.4 | 0 | 0 | 1 | 0.3 | 4 | 1.1 |
| **Hypoglycemia** | 1 | 0.3 | 1 | 0.4 | 2 | 2.1 | 3 | 0.8 | 4 | 1.1 |
| **Hypotension** | 3 | 0.8 | 1 | 0.4 | 0 | 0 | 1 | 0.3 | 4 | 1.1 |
| **Acute kidney injury** | 1 | 0.3 | 1 | 0.4 | 1 | 1.0 | 2 | 0.6 | 3 | 0.8 |
| **Constipation** | 1 | 0.3 | 1 | 0.4 | 1 | 1.0 | 2 | 0.6 | 3 | 0.8 |
| **Gastroenteritis** | 1 | 0.3 | 1 | 0.4 | 1 | 1.0 | 2 | 0.6 | 3 | 0.8 |
| **Muscle spasms** | 2 | 0.5 | 1 | 0.4 | 0 | 0 | 1 | 0.3 | 3 | 0.8 |
| **Edema peripheral** | 2 | 0.5 | 1 | 0.4 | 0 | 0 | 1 | 0.3 | 3 | 0.8 |
| **Rash** | 2 | 0.5 | 1 | 0.4 | 0 | 0 | 1 | 0.3 | 3 | 0.8 |
| **Alanine aminotransferase increased** | 0 | 0 | 2 | 0.8 | 0 | 0 | 2 | 0.6 | 2 | 0.5 |
| **Aspartate aminotransferase increased** | 0 | 0 | 2 | 0.8 | 0 | 0 | 2 | 0.6 | 2 | 0.5 |
| **Cardiac failure** | 1 | 0.3 | 0 | 0 | 1 | 1.0 | 1 | 0.3 | 2 | 0.5 |
| **Conjunctivitis** | 0 | 0 | 2 | 0.8 | 0 | 0 | 2 | 0.6 | 2 | 0.5 |
| **Eczema** | 1 | 0.3 | 1 | 0.4 | 0 | 0 | 1 | 0.3 | 2 | 0.5 |
| **Hyperuricemia** | 0 | 0 | 2 | 0.8 | 0 | 0 | 2 | 0.6 | 2 | 0.5 |
| **Myalgia** | 0 | 0 | 1 | 0.4 | 0 | 0 | 1 | 0.3 | 2 | 0.5 |
| **Palpitations** | 1 | 0.3 | 0 | 0 | 0 | 0 | 0 | 0 | 2 | 0.5 |
| **Nausea** | 1 | 0.3 | 0 | 0 | 1 | 1.0 | 1 | 0.3 | 2 | 0.5 |
| **Patients with any TRAEs** | **19** | **5.0** | **12** | **4.7** | **2** | **2.1** | **14** | **3.9** | **32** | **8.4** |
| **Dizziness** | 9 | 2.4 | 2 | 0.8 | 0 | 0 | 2 | 0.6 | 10 | 2.6 |
| **Hyperkalaemia** | 0 | 0 | 6 | 2.3 | 0 | 0 | 6 | 1.7 | 6 | 1.6 |
| **Hypotension** | 2 | 0.5 | 1 | 0.4 | 0 | 0 | 1 | 0.3 | 3 | 0.8 |
| **Headache** | 1 | 0.3 | 0 | 0 | 0 | 0 | 0 | 0 | 2 | 0.5 |
| **Diarrhea** | 0 | 0 | 1 | 0.4 | 1 | 1.0 | 2 | 0.6 | 2 | 0.5 |

Abbreviations: AE, adverse event; AZL-M, azilsartan medoxomil; TEAE, treatment-emergent adverse event; TRAE, treatment-related adverse event.
