# Supplementary material for: A Prospective Study of Azilsartan Medoxomil in the Treatment of Patients with Essential Hypertension and Type 2 Diabetes in Asia
Source: Int J Hypertens. 2022 Jan 7;2022:2717291. doi: 10.1155/2022/2717291 (PMC8759883; doi:10.1155/2022/2717291)
Supplement: Supplementary Materials — The supplementary materials consist of three files, two tables (Supplementary Table 1, listing the common adverse events reported in the study, and Supplementary Table 2, summarizing results from previous studies conducted with AZL-M) and one figure (Supplementary Figure 1, displaying the change from baseline in trough sitting SBP and DBP in mm Hg). All files have been submitted in MS Word format. [file 2717291.f1.zip › 2717291.f1/Supplementary Table 2_16th Jan '20.docx]

Supplementary Table 2. Summary results from previous studies with AZL-M

| **Study** | **Reference** | **Region/country** | **Response rates at 6 weeks by dose of AZL-M** |
| --- | --- | --- | --- |
| Phase 3, double-blind, randomized controlled study to evaluate AZL-M 20, 40, and 80 mg (compared to olmesartan medoxomil and placebo) in adult patients with essential hypertension | Bakris 2011^16^; NCT00696241^25^ | United States and Latin America | 45.3% (40-mg dose)  52.0% (80-mg dose) |
| Phase 3, double-blind, randomized controlled study to evaluate AZL-M 40 and 80 mg (compared to olmesartan, valsartan and placebo) in adult patients with essential hypertension | White 2011^26^; NCT00696436^27^ | United States and Latin America | 49.1% (40-mg dose)  52.6% (80-mg dose) |
| Phase 3, double-blind, randomized controlled study to evaluate AZL-M 40, and 80 mg (compared to placebo) in adult patients with essential hypertension | Juhasz 2918^20^ | South Korea | 62.2% (40-mg dose)  65.9% (80-mg dose) |

Abbreviations: AZL-M, azilsartan medoxomil.
